# Supplementary figures and images for: Ambient air pollutant mixture and lung function among children in Fresno, California
Source: PLoS One. 2025 Oct 31;20(10):e0335731. doi: 10.1371/journal.pone.0335731 (PMC12578181; doi:10.1371/journal.pone.0335731)

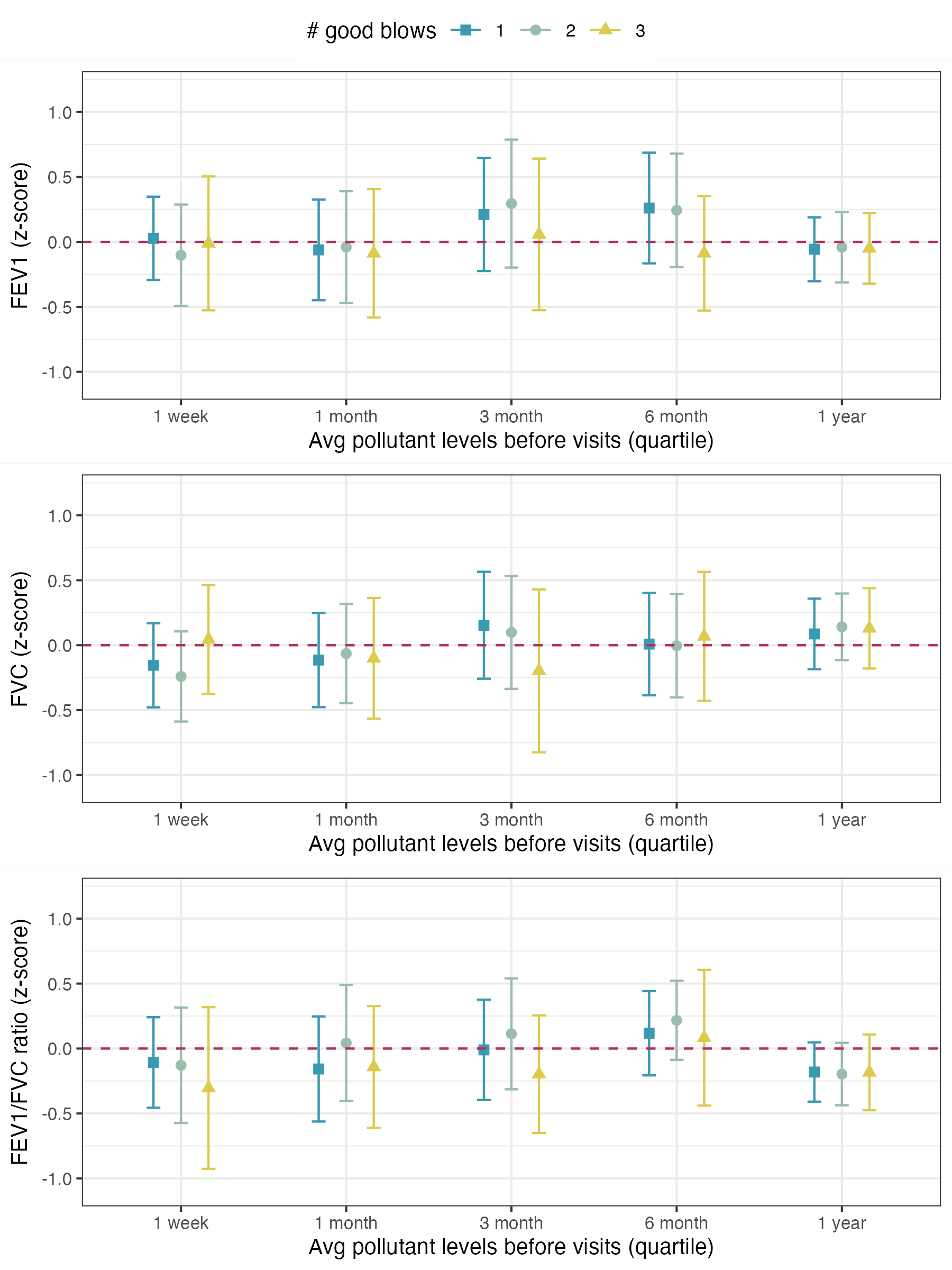

Supplement: S1 Fig — Note: All models adjusted for the sufficient adjustment set (season, neighborhood SES, race/ethnicity as a proxy for structural racism, and household SES) and applied IPCW. Cluster-based bootstrapping was used to account for repeated measures. Setting repeatability criteria of at least 1, 2, or 3 good spirometry blows at both visits restricted the analyses to 454, 384, and 214 observations, respectively. (TIFF) [file pone.0335731.s001.tiff]

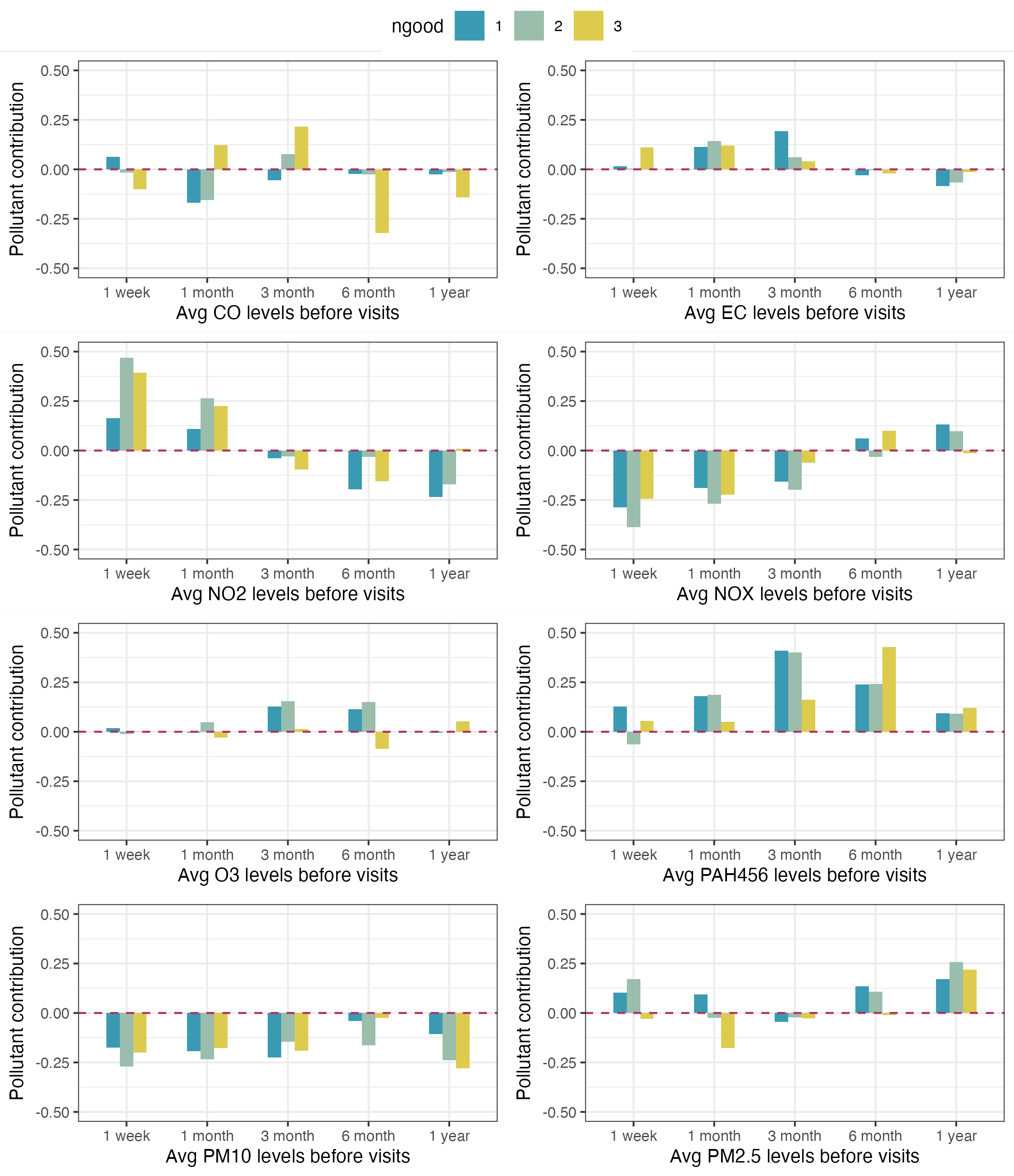

Supplement: S2 Fig — Note: The point estimates and confidence intervals of the same q-gcomp models are summarized in Figure S2. Pollutant contribution weights for the same model (same exposure time frame and repeatability criteria) are directly comparable. Negative weights represent negative contributions (harmful) to the lung function outcome. (TIFF) [file pone.0335731.s002.tiff]

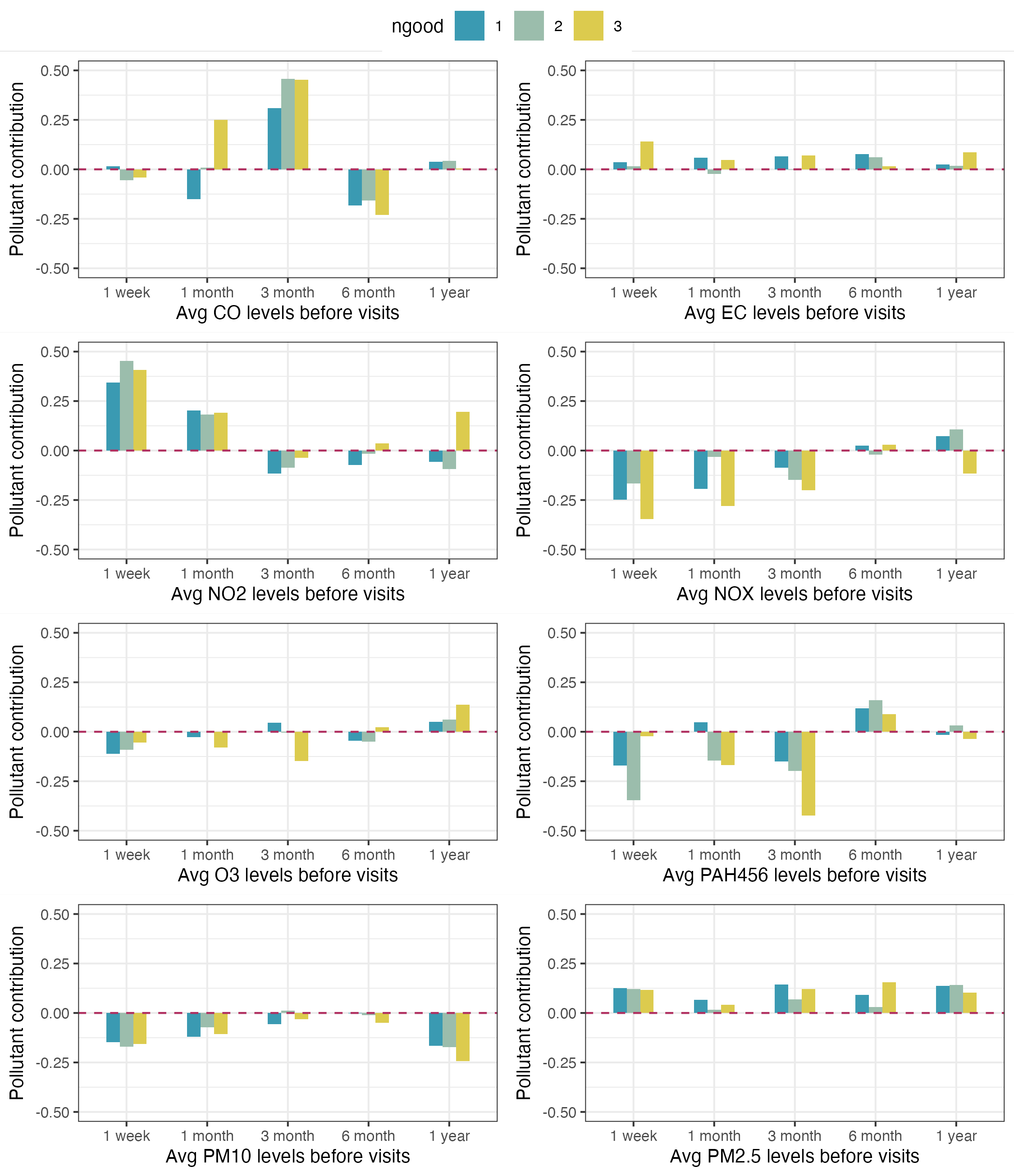

Supplement: S3 Fig — Note: The point estimates and confidence intervals of the same q-gcomp models are summarized in Figure S2. Pollutant contribution weights for the same model (same exposure time frame and repeatability criteria) are directly comparable. Negative weights represent negative contributions (harmful) to the lung function outcome. (TIFF) [file pone.0335731.s003.tiff]

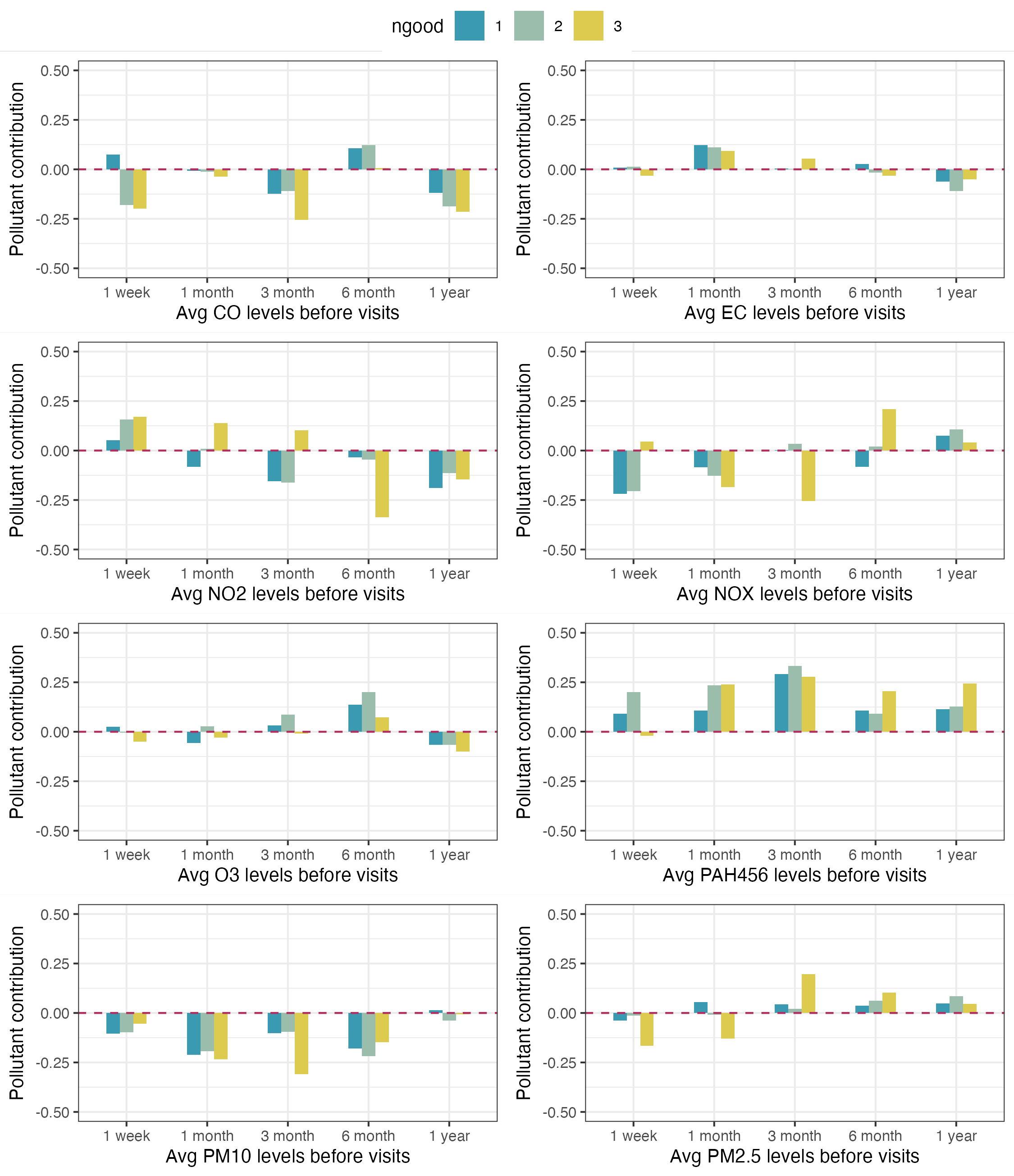

Supplement: S4 Fig — Note: The point estimates and confidence intervals of the same q-gcomp models are summarized in Figure S2. Pollutant contribution weights for the same model (same exposure time frame and repeatability criteria) are directly comparable. Negative weights represent negative contributions (harmful) to the lung function outcome. (TIFF) [file pone.0335731.s004.tiff]

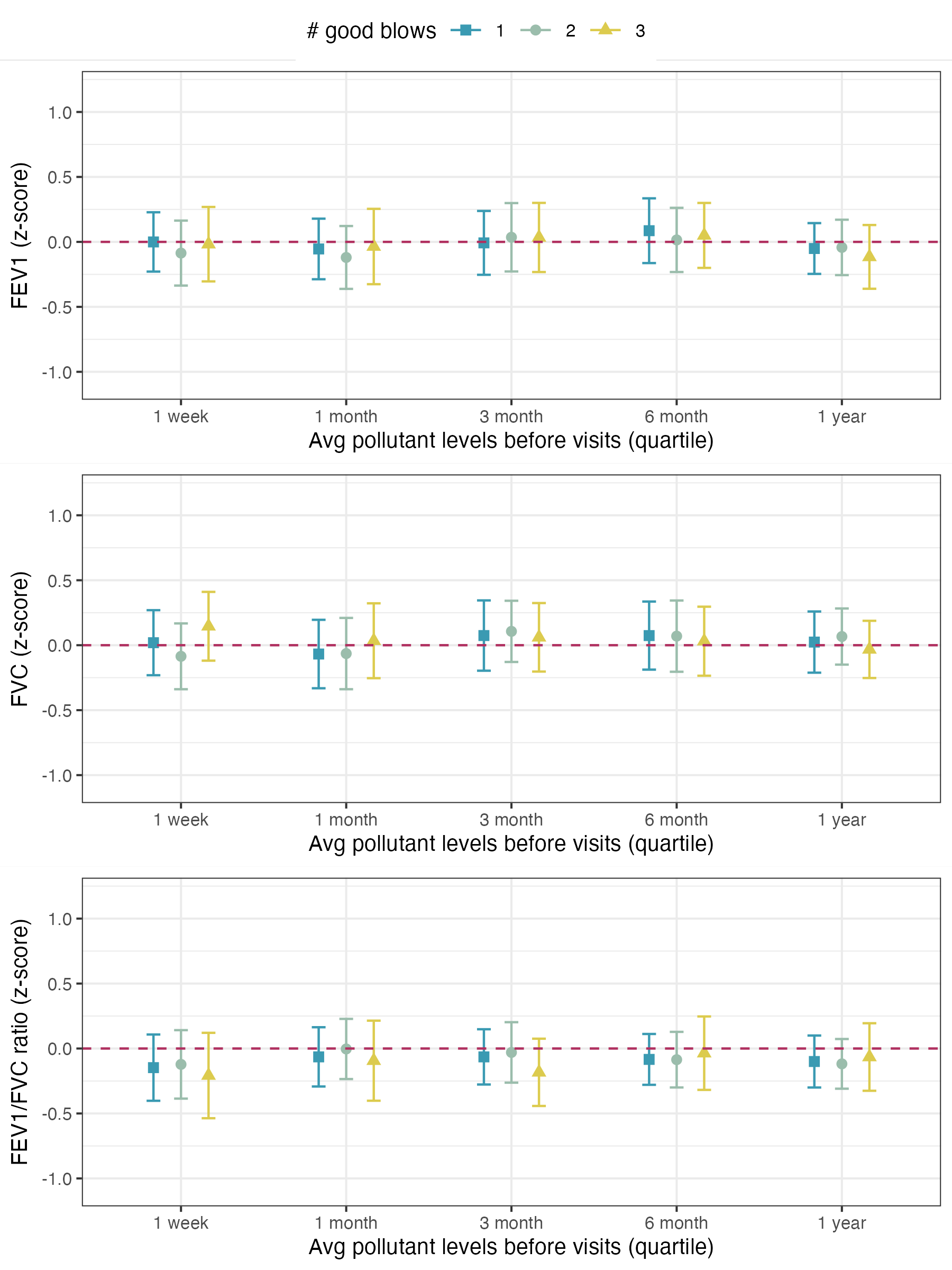

Supplement: S5 Fig — Note: All models adjusted for the sufficient adjustment set (season, neighborhood SES, race/ethnicity as a proxy for structural racism, and household SES) and applied IPCW. Cluster-based bootstrapping was used to account for repeated measures. Setting repeatability criteria of at least 1, 2, or 3 good spirometry blows at both visits restricted the analyses to 454, 384, and 214 observations, respectively. Seven ambient air pollutants, except for ozone, are analyzed. The results for the same analyses with eight air pollutants, including ozone, are shown in Figure S2. (TIFF) [file pone.0335731.s005.tiff]
