# Supplementary material for: Ambient air pollutant mixture and lung function among children in Fresno, California
Source: PLoS One. 2025 Oct 31;20(10):e0335731. doi: 10.1371/journal.pone.0335731 (PMC12578181; doi:10.1371/journal.pone.0335731)
Supplement: S1 File — (PDF) [file pone.0335731.s006.pdf]

File S1: Directed acyclic graph characterizing the causal pathways between ambient air pollution and children's lung function

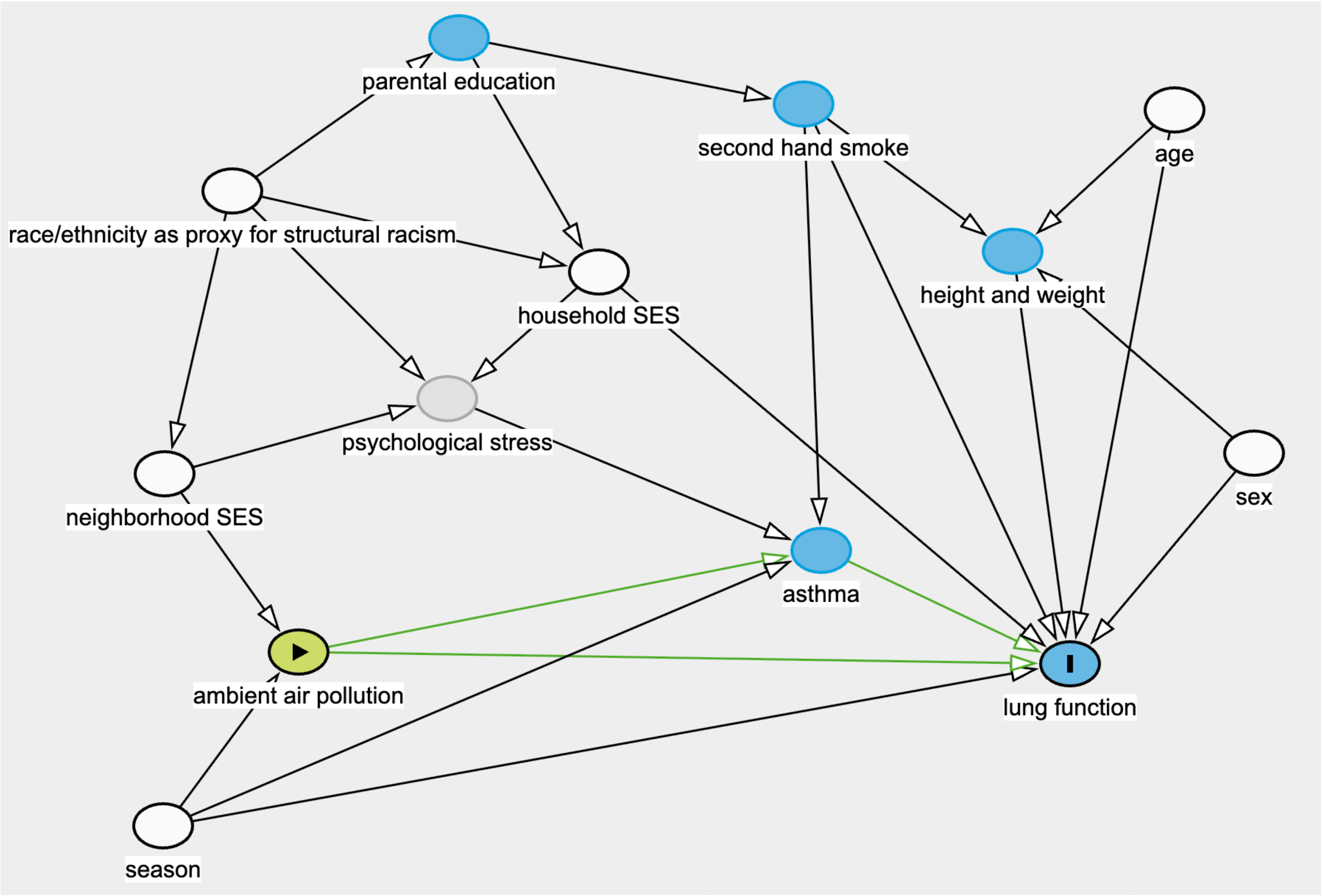

Note: The green node represents the exposure variable, the white nodes represent variables adjusted in the sufficient adjustment set. The blue nodes represent outcome variables and variables associated with the outcomes. The grey nodes represent the unobserved variables. Arrows represent causal pathways. Age and sex were accounted for when standardizing lung function measurements. Asthma status is a mediator between ambient air pollution exposure and lung function and should not be adjusted in statistical analyses.
