# Supplementary material for: Ambient air pollutant mixture and lung function among children in Fresno, California
Source: PLoS One. 2025 Oct 31;20(10):e0335731. doi: 10.1371/journal.pone.0335731 (PMC12578181; doi:10.1371/journal.pone.0335731)
Supplement: S2 File — (PDF) [file pone.0335731.s007.pdf]

File S2: The causal pathways of selection bias due to differential censoring at the second visit in the CHAPS study

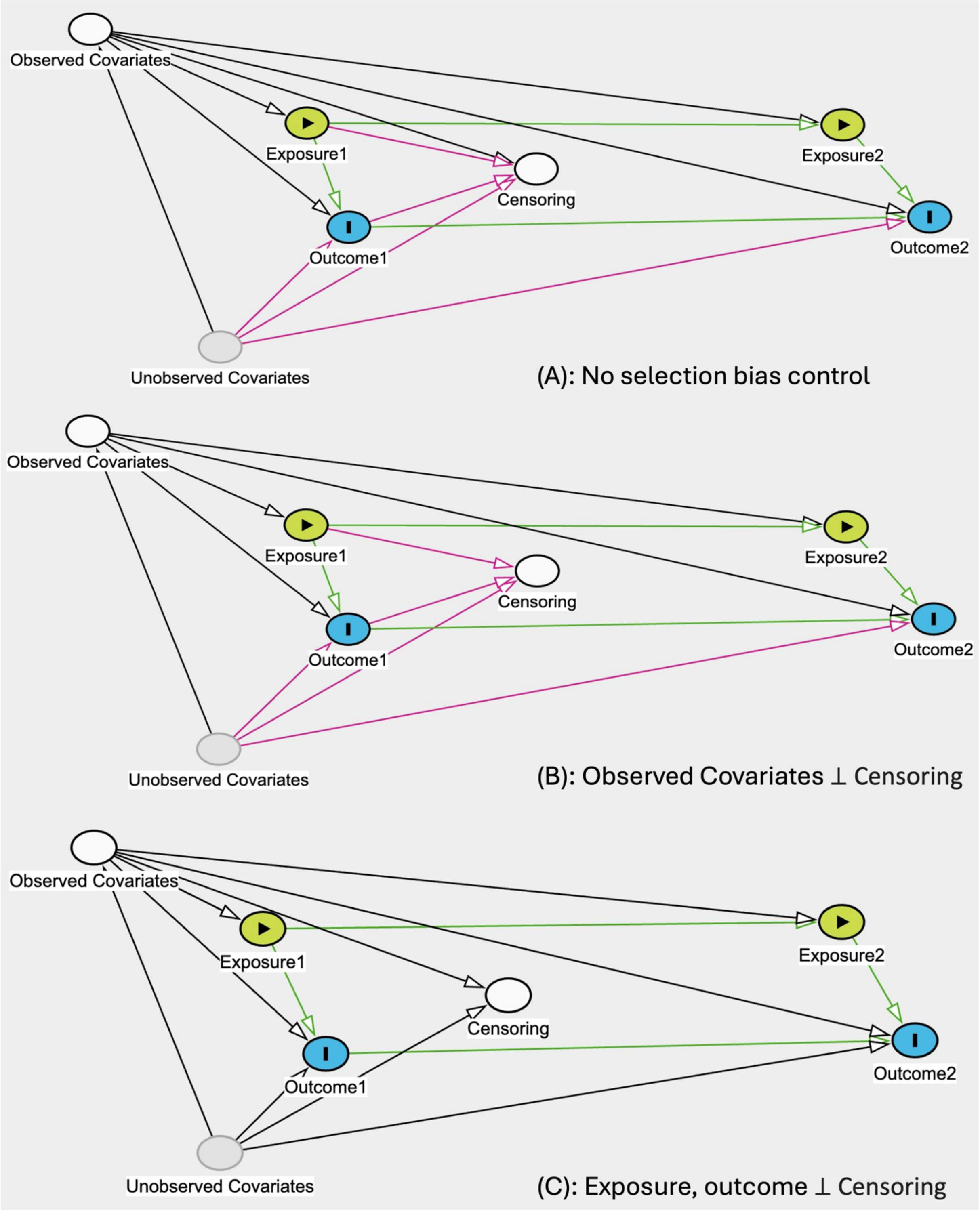

Note: The green node represents the exposure variable. The blue node represents the outcome variable. The white nodes represent observed and controlled variables. The grey node represents unobserved variables. The arrows represent causal pathways.

Censoring, or not completing the second visit, may be associated with several preceding factors, such as observed covariates, unobserved covariates, exposure at the first visit, and outcome at the first visit. We assume that after controlling for the sufficient adjustment set identified in **Figure S1**, there is no residual confounding by unobserved covariates, illustrated by the absence of an arrow between unobserved covariates and exposures in the figure in **File S1**.

S1 (A) illustrates the scenario where observed covariates are controlled, but no additional method is applied to address selection bias. There are several open causal pathways for selection bias on the relationship between exposure 2 and outcome 2:

Exposure 2 ← Exposure 1 → [Censoring] ← Unobserved Covariate → Outcome 1 → Outcome 2

Exposure 2 ← Exposure 1 → [Censoring] ← Unobserved Covariate → Outcome 2.

Exposure 2 ← Exposure 1 → [Censoring] ← Outcome 1 → Outcome 2.

Exposure 2 ← Exposure 1 → Outcome 1 → [Censoring] ← Unobserved Covariate → Outcome 2.

S1 (B) illustrates the scenario where an additional inverse probability of censoring weights (IPCW) was applied to break the association between observed covariates and censoring. In this scenario, the open selection bias pathways related to exposure at the first visit persisted. In other words, the selection bias pathway related to observed covariates (E.g., Exposure 2 ← Exposure 1 → [Censoring] ← Observed Covariate → Outcome 2) is already blocked when the observed covariates are adjusted. Applying additional IPCW based on observed covariates does not further reduce bias.

S1 (C) illustrates the scenario where observed covariates are controlled, and an IPCW was applied to break the association between exposure and outcome at the first visit and censoring. Under the assumptions that unmeasured confounding is independent of exposure given observed covariates, all biasing pathways are blocked, and the causal relationship between air pollution exposure and child asthma symptoms can be identified.

The IPCW is calculated as $1/\widehat{Pr}(C = 0|E = e, O = o)$, which is the inverse of the conditional probability of remaining in the study at the second visit, given the participant's observed exposure and outcome at the first visit, estimated from a logistic regression. By applying this weight, children who were more likely to be censored were up-weighted, while children who were more likely to remain in the study were down-weighted. Applying this IPCW created a pseudo-population where censorship status at the second visit was independent of exposure and outcome status and broke the causal pathway for selection bias. We calculated the IPCW using the 12-month exposures to the 7 pollutants, except for ozone, and the children's FEV1 levels at the first visit for higher data completeness.
