# Supplementary material for: Ambient air pollutant mixture and lung function among children in Fresno, California
Source: PLoS One. 2025 Oct 31;20(10):e0335731. doi: 10.1371/journal.pone.0335731 (PMC12578181; doi:10.1371/journal.pone.0335731)

**File S3**: Correlation plots for the Children's Health and Air Pollution Study (CHAPS) participants' average residential exposures to eight ambient air pollutants before visits.

(1): Correlation plot of 1-week average exposures to eight ambient air pollutants

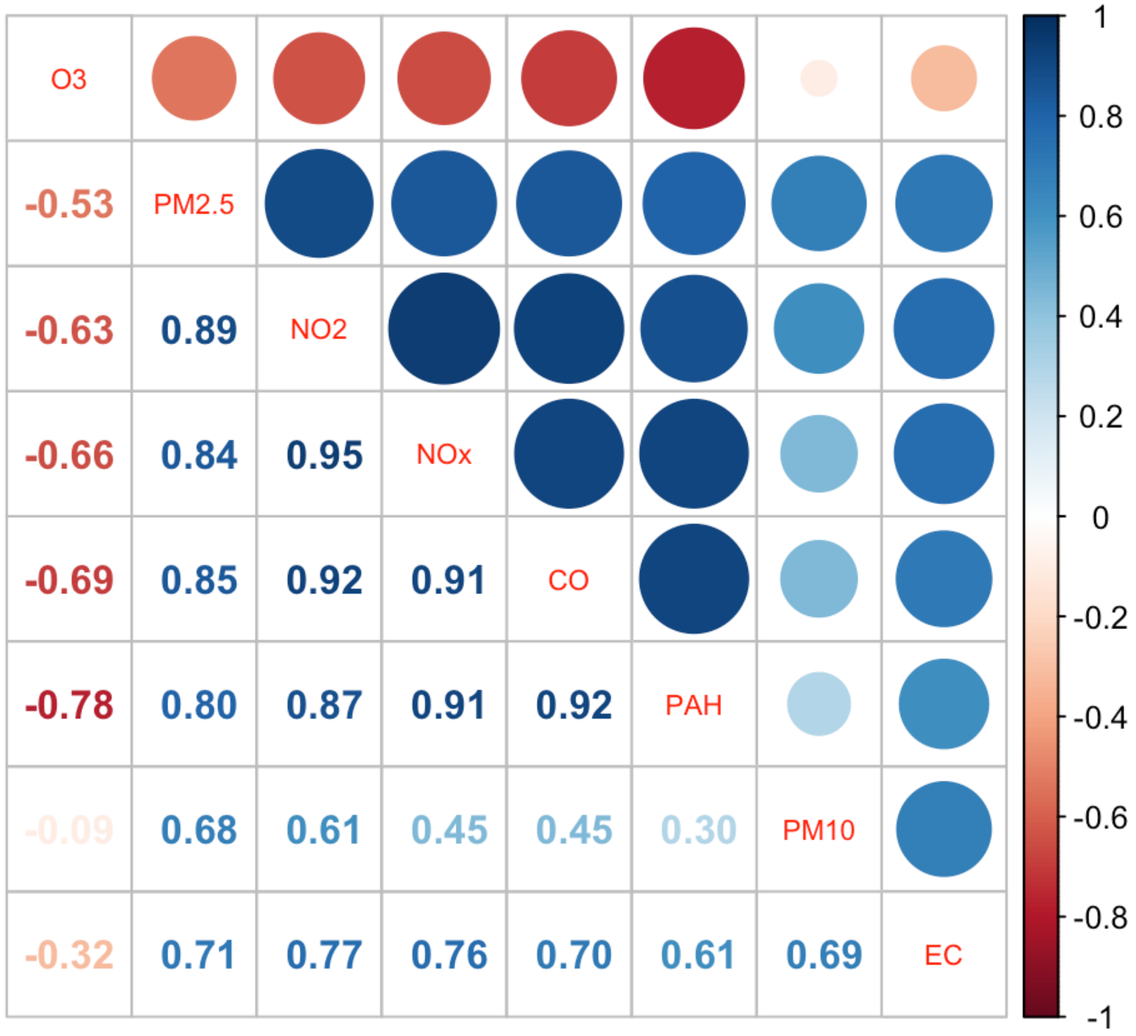

(2): Correlation plot of 1-month average exposures to eight ambient air pollutants

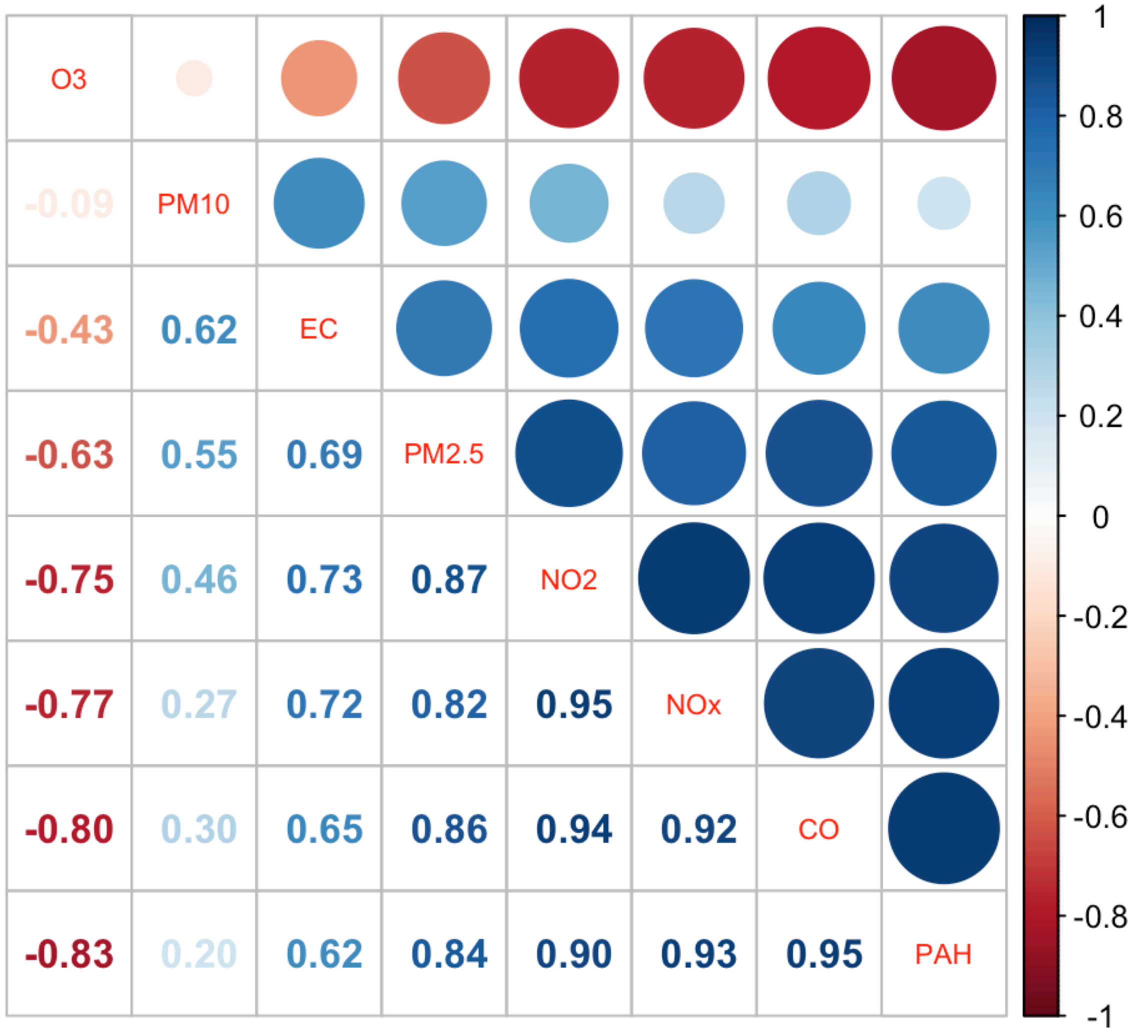

(3): Correlation plot of 3-month average exposures to eight ambient air pollutants

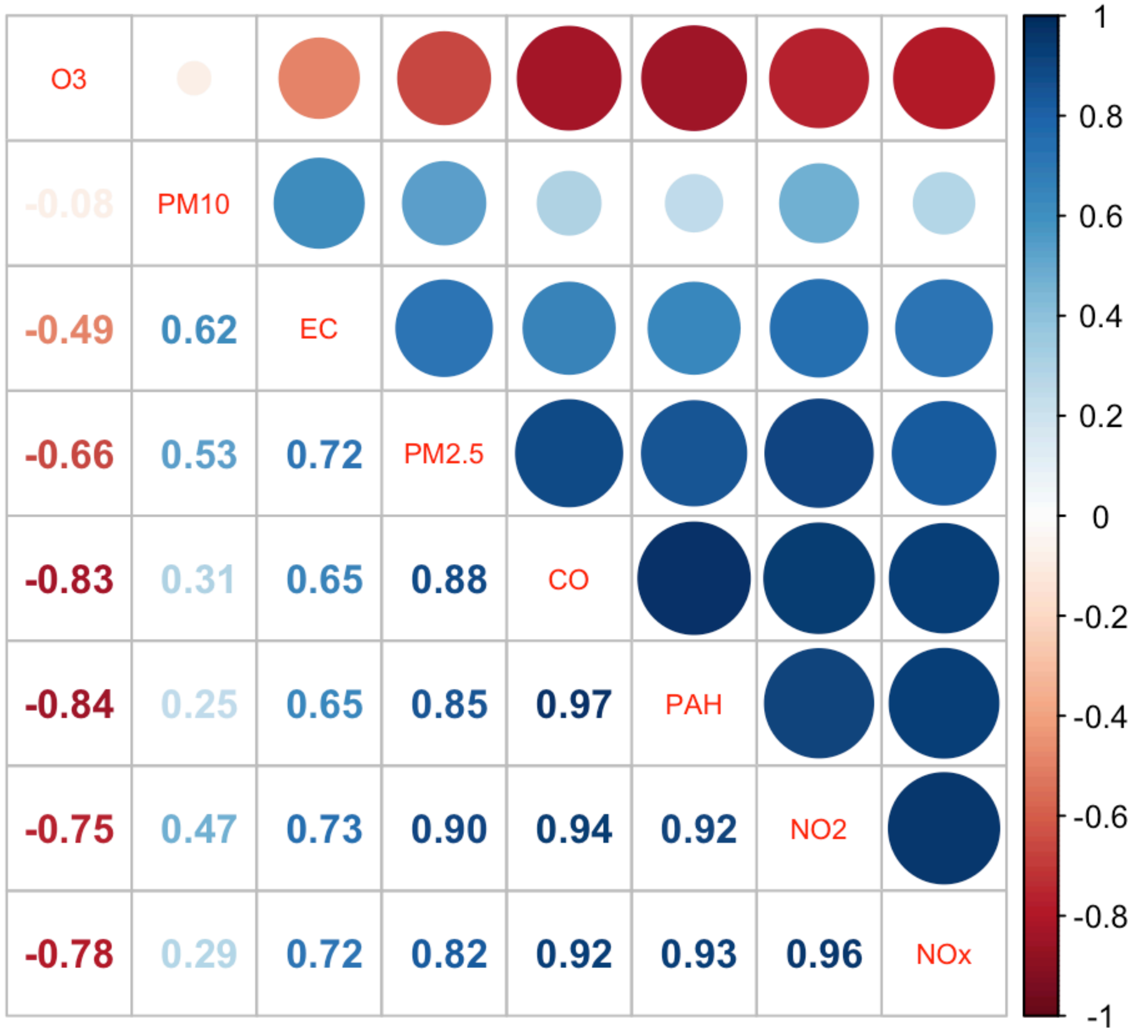

(4): Correlation plot of 6-month average exposures to eight ambient air pollutants

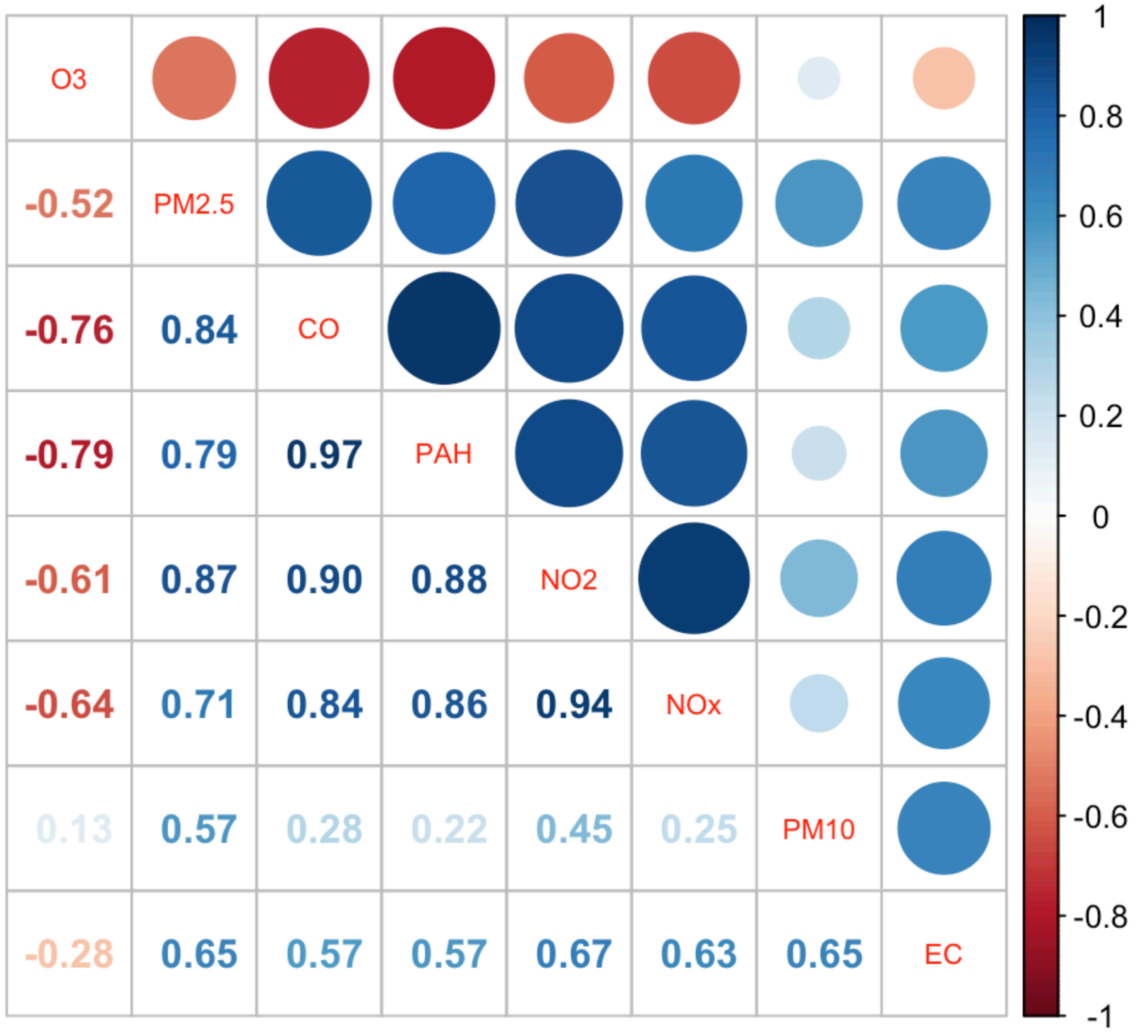

(5): Correlation plot of 1-year average exposures to eight ambient air pollutants

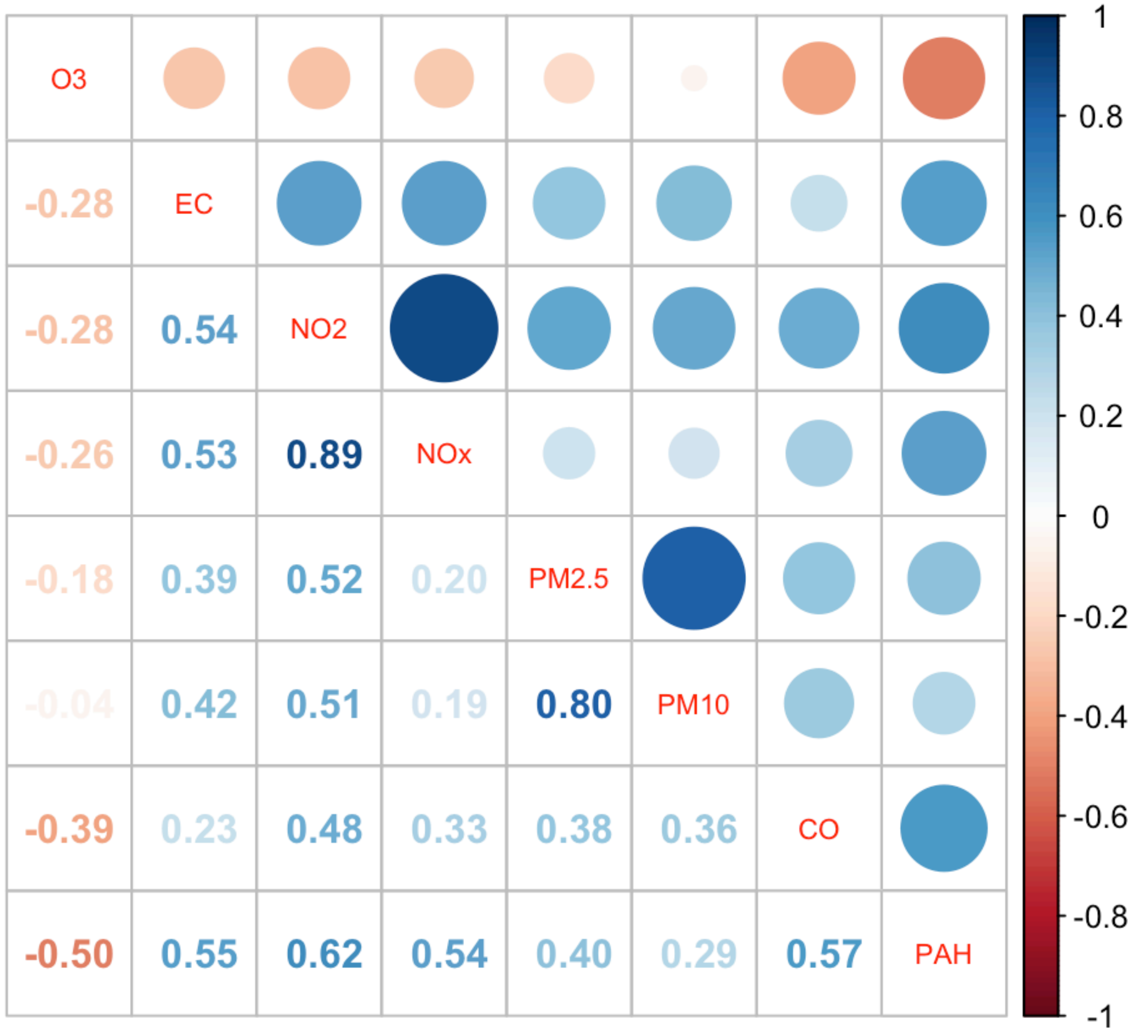

Supplement: S3 File — (PDF) [file pone.0335731.s008.pdf]
