# Supplementary material for: Ambient air pollutant mixture and lung function among children in Fresno, California
Source: PLoS One. 2025 Oct 31;20(10):e0335731. doi: 10.1371/journal.pone.0335731 (PMC12578181; doi:10.1371/journal.pone.0335731)
Supplement: Attachment — Submitted filename: Review.pdf [file pone.0335731.s009.pdf]

1. Summary of the research

This manuscript investigates the relationships between exposures to eight ambient air pollutants (PM2.5, PM10, NO2, NOx, CO, O3, EC, and PAH456) and lung function among 299 children aged 7–9 years in Fresno, California, a disadvantaged community with high ambient air pollution. Using both traditional single-pollutant linear mixed-effect models and quantile-based g-computation (q-gcomp) for mixture analysis, the study identifies PM10 as the key contributor associated with lower standardized FEV1 and FEV1/FVC ratio, particularly for 3-month average exposures.

The study is novel in applying a mixture analysis method that accounts for correlations among multiple pollutants and evaluates different exposure timeframes, which extends prior research that focused on a limited set of pollutants or single-pollutant models. Strengths include high-quality spirometry with repeatability criteria, the application of GLI race-neutral calculators, rigorous adjustment for confounders via a DAG, and sensitivity analyses using inverse probability of censoring weights (IPCW). Weaknesses include some figure clarity issues, limited reporting of model parameters for q-gcomp, and reliance on residential exposure estimates rather than personal monitoring. Overall, this is a well-conceived and valuable study that could be suitable for publication after addressing the following points.

2. Examples and evidence

Major issues (must be addressed for the manuscript to proceed)

- **Figure clarity**: Many figures (Figures 1–5, S3–S8) have blurry axis labels and tick marks. Improving resolution and label readability is necessary.
- **Model details**: Provide more explicit details about q-gcomp model parameters, including the number of quantiles, bootstrapping settings, and handling of O3 correlations, to allow reproducibility by other researchers.

Minor issues (suggested improvements)

- **Code and data sharing**: Make the R scripts and related datasets available in an open repository (e.g., GitHub), if possible, to enhance transparency and reproducibility.
- **Figures and tables**: Consider combining or simplifying supplemental figures (S5–S8) for clarity and ease of interpretation.
- **Results validation**: Include analyses with and without IPCW (or discuss explicitly if already done) to show the robustness of the findings to potential selection bias.
- **Text clarity**: Minor language and formatting improvements could enhance readability, particularly in the Results and Discussion sections.

- **Interpretation of q-gcomp weights**: Provide guidance for interpreting variability in pollutant weights and how they relate to single-pollutant estimates, highlighting limitations due to categorization and correlation structure.
- **Exposure assessment limitations**: Clarify the potential impact of relying on residential exposures and not capturing school or indoor exposures, and discuss implications for effect estimates.

3. Other points (optional)

Confidential to Editor: None. I am available to review a revised version.
